# Colposcopy non-attendance following an abnormal cervical cancer screening result: a prospective population-based cohort study

**DOI:** 10.1186/s12905-022-01851-6

**Published:** 2022-07-09

**Authors:** Linda A. Liang, Sylke R. Zeissig, Gunther Schauberger, Sophie Merzweiler, Kathrin Radde, Sabine Fischbeck, Hans Ikenberg, Maria Blettner, Stefanie J. Klug

**Affiliations:** 1 Epidemiology, Department of Sports and Health Sciences, Technical University of Munich, Georg-Brauchle-Ring 56, 80992 Munich, Germany; 2 Institute for Clinical Epidemiology and Biometry, Julius-Maximilians University of Würzburg, Würzburg, Germany; 3 Department of Psychosomatic Medicine and Psychotherapy, Medical Psychology and Medical Sociology, University Medical Center, Johannes Gutenberg University of Mainz, Mainz, Germany; 4 Cytomol MVZ, Frankfurt, Germany; 5 Institute of Medical Biostatistics, Epidemiology and Informatics, University Medical Center, Johannes Gutenberg University of Mainz, Mainz, Germany

**Keywords:** Colposcopy, Non-attendance, Screening follow-up, Abnormal screening result, Cervical cancer screening, HPV status, HPV testing

## Abstract

**Background:**

A considerable proportion of cervical cancer diagnoses in high-income countries are due to lack of timely follow-up of an abnormal screening result. We estimated colposcopy non-attendance, examined the potential factors associated and described non-attendance reasons in a population-based screening study.

**Methods:**

Data from the MARZY prospective cohort study were analysed. Co-test screen-positive women (atypical squamous cells of undetermined significance or worse [ASC-US+] or high-risk human papillomavirus [hrHPV] positive) aged 30 to 65 years were referred to colposcopy within two screening rounds (3-year interval). Women were surveyed for sociodemographic, HPV-related and other data, and interviewed for non-attendance reasons. Logistic regression was used to examine potential associations with colposcopy attendance.

**Results:**

At baseline, 2,627 women were screened (screen-positive = 8.7%), and 2,093 again at follow-up (screen-positive = 5.1%; median 2.7 years later). All screen-positives were referred to colposcopy, however 28.9% did not attend despite active recall. Among co-test positives (ASC-US+ and hrHPV) and only hrHPV positives, 19.6% were non-attendees. Half of only ASC-US+ screenees attended colposcopy. Middle age (adjusted odds ratio [aOR] = 1.55, 95% CI 1.02, 4.96) and hrHPV positive result (aOR = 3.04, 95% CI 1.49, 7.22) were associated with attendance. Non-attendance was associated with having ≥ 3 children (aOR = 0.32, 95% CI 0.10, 0.86). Major reasons for non-attendance were lack of time, barriers such as travel time, need for childcare arrangements and the advice against colposcopy given by the gynaecologist who conducted screening.

**Conclusions:**

Follow-up rates of abnormal screening results needs improvement. A systematic recall system integrating enhanced communication and addressing follow-up barriers may improve screening effectiveness.

**Supplementary Information:**

The online version contains supplementary material available at 10.1186/s12905-022-01851-6.

## Background

Cervical cancer (CC) is preventable with effective primary and secondary prevention measures such as human papillomavirus (HPV) vaccination and screening. Cervical cancer screening (CCS) includes cytological assessment, viral detection of HPV or both (co-testing) [1]. However, following an abnormal screening result where risk of progression to CC is elevated, colposcopy is an important step to guide management [2]. Colposcopy involves magnified visual inspection of the cervix and biopsy extraction where necessary by trained and experienced colposcopists. Non-adherence to follow-up of abnormal screening results, i.e. colposcopy non-attendance, may lead to undiagnosed precancer (cervical intraepithelial neoplasia, CIN) and preventable CC [3], undermining screening effectiveness [4].

Until 2020, Germany offered free opportunistic Pap screening annually to women from age 20, but quality assurance measures were not systematically monitored [5]. Despite reasonable coverage [6] and declines in incidence, up to half of invasive CC cases were diagnosed in women screened frequently in the preceding 10 years [7]. Over two thirds of diagnoses had preceding negative screening results [8]. Failure of CCS to detect disease include sample collection issues to detect abnormal cells, but also lack of follow-up after an abnormal screening result [9]. The latter is not unique to Germany. For example in the US, 8% of CC diagnoses were attributed to colposcopy non-attendance [10] and a meta-analysis attributed 12% of CC to poor follow-up care [11]. Follow-up failures can be minimised if referrals are part of a failsafe recall system, via systemic tracking, call-and-recall invitations and reminders [2, 12]. In 2020, HPV testing was adopted as a co-test in women 35 years of age and older in Germany [5]. Therefore, it is important to identify sub-groups likely to be non-adherent with follow-up, particularly with the addition of HPV screening.

Several studies have examined potential factors associated with colposcopy non-attendance [3, 10, 13–23]. However, most lack individual socio-demographic information [10, 13, 16, 17, 20], or are based on underserved populations such as migrants [13, 14]. The role of HPV status on follow-up attendance was explored only recently in a small pilot study [24]. Additionally, small qualitative studies have examined reasons for colposcopy non-attendance [25, 26]. We estimated colposcopy non-attendance among screen-positive women from a population-based, real-world screening study involving co-testing and examined the potential factors associated with attendance. Additionally, we described non-attendance reasons.

## Methods

### Participants and data collection

The data stem from randomly recruited participants from the general population (n = 2,627) who were screened within the randomised trial and prospective cohort MARZY study, described previously [27, 28]. Briefly, women eligible from the general population (aged 30 to 65 years, with no history of hysterectomy or CC and not pregnant) were screened by office-based gynaecologists at study baseline (R1, 2005–2007) with routine Pap smear, plus an additional MARZY study swab (liquid-based cytology, ThinPrep, Cytyc/Hologic including subsequent HPV testing, Hybrid Capture®2). HPV co-testing was investigated [27]. Participants were administered a questionnaire (Q1) relating to sociodemographic and other factors.

**Fig. 1:**
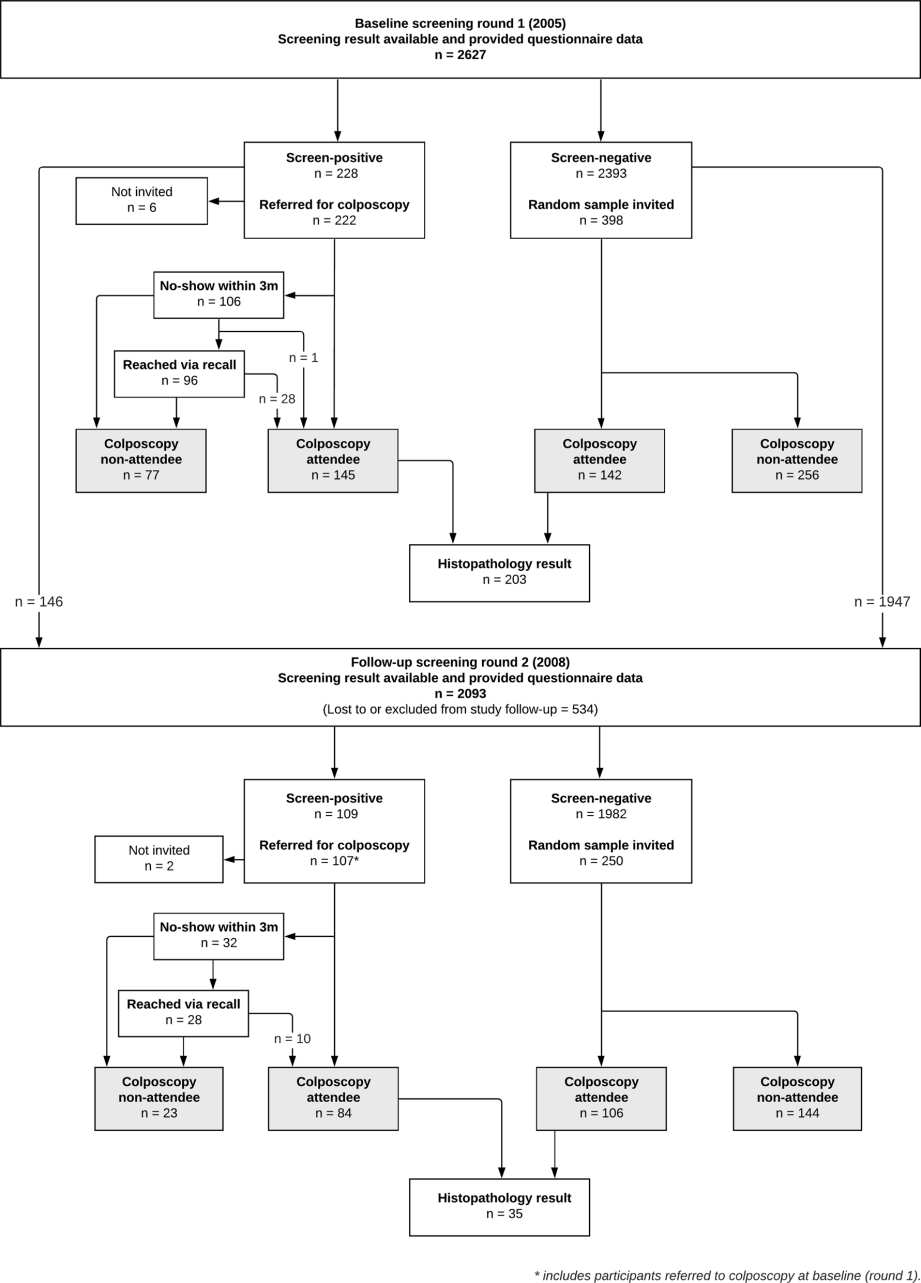
Flowchart of screening referrals and their screening results

Positive screening results were defined as atypical squamous cells of undetermined significance or worse (ASC-US+) or high-risk HPV positive (hrHPV). Screen-positives were contacted by postal letter, which included HPV information and referred to the study colposcopy clinic (University Medical Center, Mainz; Fig. 1). These letters contained additional information on HPV infection and explained the colposcopy procedure in simple terms. Active telephone recall efforts were carried out by female study personnel to improve colposcopy attendance rates among women who did not arrange an appointment at the study clinic within 3 months of referral. Personnel also interviewed non-attendees for their reasons on non-attendance.

Screening was conducted again 3 years later (R2, 2008–2010) among women who participated in R1 and were still eligible (no hysterectomy or CC diagnoses since R1 and not pregnant). Lifestyle exposures such as smoking status were updated in a second questionnaire (Q2). Active recalls were again conducted by female study personnel if screen-positive participants had not attended colposcopy within 3 months following referral to the designated study clinic (University Medical Center, Mainz and St. Vincenz and Elisabeth Hospital, Mainz).

After R2 concluded (2010), an additional questionnaire with HPV-related questions (Q3) was administered to all hrHPV positive women, investigating perception and communication of HPV results, and HPV knowledge. As the MARZY screenings were conducted with a 3-year interval but routine Pap screenings were offered opportunistically and annually in the study region, any screenings conducted outside the study between the two MARZY rounds were retrospectively documented.

### Colposcopy -attendance

We classified colposcopy attendance status using medical records from the designated colposcopy clinics. The primary outcome was non-attendance after referral to colposcopy within a 4 month time-frame, calculated as number of non-attendees among all referrals. This definition is based on the study referral threshold (ASC-US+ or hrHPV or both positive). At the time of study conduct, the 2008 European guidelines suggested colposcopies be conducted following ASC-US+ and hrHPV positive results [9]. The German CCS guideline in effect at the time advised women with low-grade squamous intraepithelial lesions or worse (LSIL+) who were also hrHPV positive to undergo colposcopy [29]. Attendance was estimated for both thresholds (ASC-US+ and hrHPV positive; LSIL+ and hrHPV positive).

### Variables of interest

Sociodemographic variables obtained included age, region of residency, nationality, highest education level attained (lower secondary; upper secondary and further), employment situation, net monthly household income (low income ≤ 1500€; higher income > 1500€), marital status, parity (≤ 2 children; ≥ 3 children) and health insurance status. Smoking status, oral contraception use and hormone replacement therapy (HRT) were dichotomised (ever vs. never). Self-reported frequency of CCS attendance was grouped (regularly every 1–2 years; irregularly every 3 years or less or never). Screen-positive was defined as ASC-US+ only, hrHPV only or both ASC-US+ and hrHPV positive, and also LSIL+ only or both LSIL and hrHPV positive.

At the time of the Q3 survey, no validated HPV knowledge scale was available for use but the questionnaire items were based on extensive review of the qualitative body of evidence published. Perceived experience during and after the screening examination and concerns about infectivity or impact on sexual relationships were based on the Psychosocial Effects of Abnormal PAP Smears Questionnaire (PEAPS-Q) [30] and Cervical Dysplasia Distress Questionnaire (CDDQ) [31]. The items of interest were sub-categorised by 5-point Likert scale or binary “yes/no” answers as (i) Perception: negative screening experience (dichotomised), degree of negative reaction and understanding regarding the positive hrHPV result such as anxiety or insecurity, and (ii) HPV knowledge: as determined by the ability to identify at least 2 areas of HPV infection (virus, persistence consequences, vaccination; dichotomised), level of HPV understanding (none to good), and prior HPV knowledge to the study. Communication (iii) that occurred between the gynaecologists and participant (dichotomised), comprehensiveness of the counselling (dedicated time, provided background information and support), trust in the physician and discussion of result between the participant and friends or family members were also analysed. Concerns (iv) regarding cancer, infertility and infectivity were captured.

### Statistical analyses

Any screen-positives leading to a referral at either round between 2005 and 2010 were included. If women were referred at both rounds, we designated questionnaire and interview data from the first referral only for regression analyses. All variables of interest were analysed using R (version 4.0.5, R Foundation for Statistical Computing, Vienna, Austria). Potential associations between attendance and individual factors were examined by univariable regression modelling and collinearity between variables were assessed. For multivariable regression, we applied multiple imputation methods to obtain model averaged estimates for missing data and computed bootstrap resampled 95% confidence intervals (CI; bootstraps = 500) using the MAMI package for R [32]. Missing data in regression models were treated as available case analyses and the adjusted odds ratios (aOR) controlled for all available confounders (age, region of residency, nationality, highest education level attained, employment situation, income, marital status, parity, smoking status, OC use, HRT use, screening frequency, screening result and insurance status), as these were previously reported to be associated with attendance [3, 10, 13–23]. Education, employment and screening result were dichotomised for regression. Non-attendee interview responses from both rounds were described together. In the case where women were non-attendees at both rounds, we designated the interview data from the first interview only. We also descriptively assessed the longitudinal outcomes (screening results, colposcopy attendance) of R1 referrals who did not attend colposcopy then but who were screened again at R2.

Informed consent was provided by all study participants prior to screening at study baseline. The MARZY study was approved by the ethical committee of the state of Rhineland-Palatinate (Landesärztekammer Rheinland-Pfalz: 837.438.03 (4100)) and the state government data protection office.

## Results

### Colposcopy attendance status

Of 2,627 women screened at R1, 228 (8.7%) were screen-positive, 222 (8.5%) were referred to colposcopy while 6 were not invited due to pre-planned hysterectomy elsewhere (Fig. 1). Initially, 106 of these 222 screen-positive women did not attend colposcopy within 3 months following referral. With active recall efforts, 96 could be reached and 28 (29.2%) attended afterwards. One woman who was not reached by telephone eventually attended colposcopy. Finally, 145 women (65.3%) attended colposcopy within 4 months, while 77 (34.7%) did not.

At R2, 2,093 (79.7%) women were screened at a median of 2.7 years later. Of the 107 screen-positive women referred to colposcopy, 32 initially did not attend after referral and 28 were reached via active recall (Fig. 1). Ten (31.3%) women were motivated to attend. Finally, 23 (21.5%) were non-attendees and 84 attended colposcopy (78.5%; Additional file 1: Table S1).

Twenty-one women were referred at both rounds where half were referred due to only hrHPV positive results (Additional file 1: Table S2). A total of 222 women (R1) and 86 women (R2) were referred to colposcopy (n = 308) in the entire study.

**Fig. 2:**
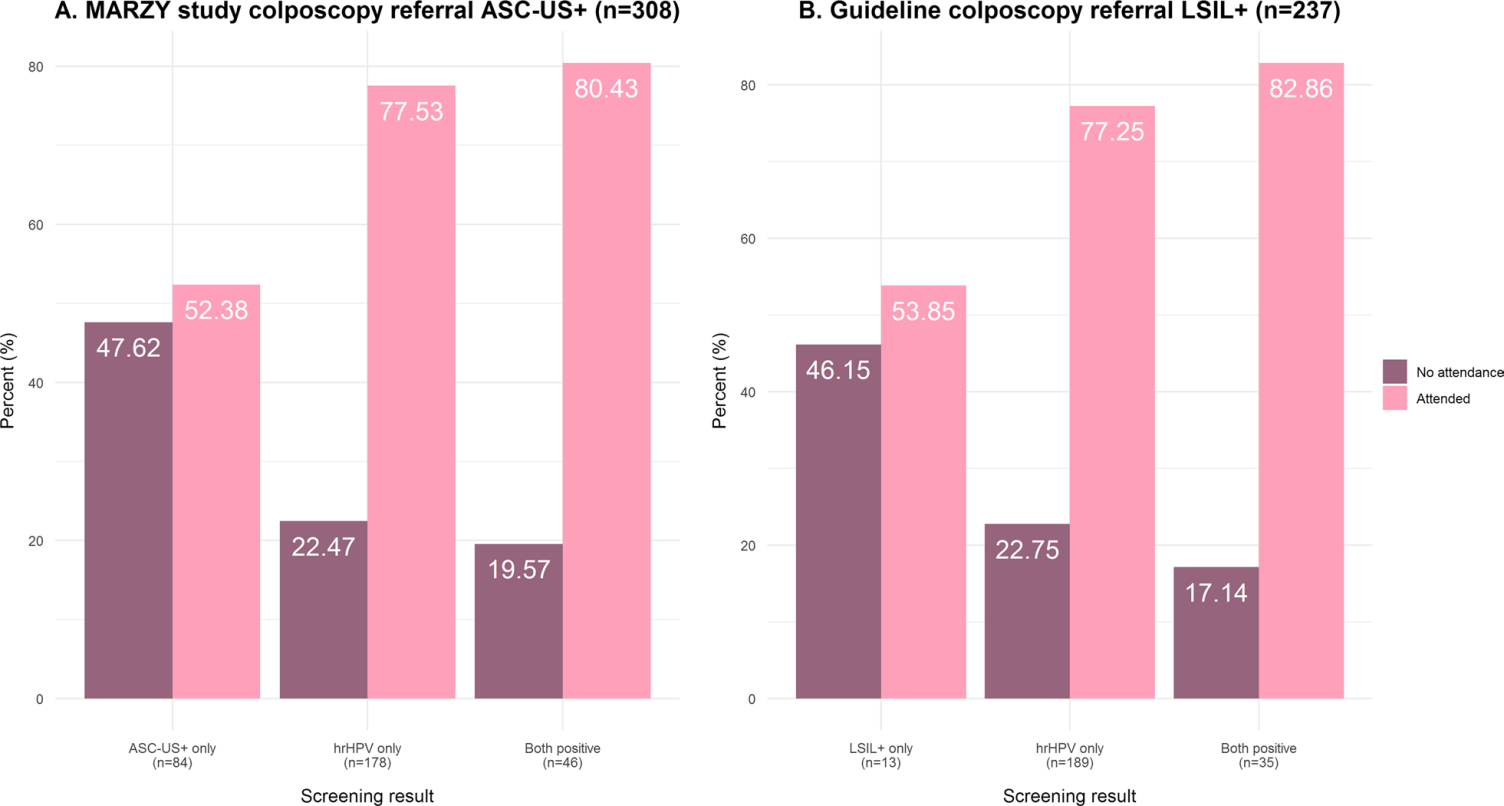
Proportion of overall referrals who attended or did not attend colposcopy by screening result at ASC-US+ threshold (**A**) and at LSIL+ (**B**). ASC-US+: Atypical squamous cells of undetermined significance or worse; hrHPV: high-risk human Papillomavirus; LSIL+: low grade squamous intraepithelial lesion or worse

Overall, among 308 total referrals, attendance was recorded in 219 (71.1%) women and non-attendance in 89 (28.9%). Mean age in both groups were similar: 45.8 years (SD = 9.1) and 45.7 years (SD = 10.1) respectively. Among both ASC-US+ and hrHPV co-test positives, 9 (19.6%) did not attend (Fig. 2A). Among LSIL+ and hrHPV positives, 6 (17.1%) did not attend colposcopy (Fig. 2B). Approximately half of only cytology-positives attended colposcopy; the majority had ASC-US (Additional file 1: Table S3). By R2, 32 women had positive routine Pap results detected between study rounds. Non-attendance rates were similar (~ 20%) after excluding these cases (Additional file 1: Figure S1).

### Sociodemographic and other factors

Compared to younger women (30–39 years), 40–49 year old women were more likely to attend colposcopy (75% vs. 69%; aOR = 1.55, 95% CI 1.02, 4.96) (Table 1). Women who resided in the urban area were less likely to attend, albeit not statistically significant (65% vs. 76%; aOR = 0.63, 95% CI 0.30, 1.00). Among women from low income households, 87% were attendees while 68% of the women from higher income households (net monthly income >1500€) attended colposcopy. Women with higher household income or who had birthed ≥ 3 children were 67% (aOR = 0.33, 95% CI 0.11, 0.92) and 68% (aOR = 0.32, 95% CI 0.10, 0.86) less likely to attend colposcopy respectively. Smoking status, oral contraceptive use and HRT were not significantly associated with attendance. Sixty percent who attended screening irregularly (every 3 years or less) or not at all, attended colposcopy versus 73% of regular participants. A positive hrHPV screening result increased likelihood of attending by threefold (aOR = 3.04, 95% CI 1.49, 7.22; Table 1).

**Table 1:** Sociodemographic and lifestyle factors associated with colposcopy attendance among all women referred

	Overall (n = 308)		Logistic regression models			
	Non-attendee (n = 89)	Attendee (n = 219)	Univariable		Multivariable^a^	
	n (row %)	n (row %)	OR	95% CI*	aOR	95% CI**
**Age group**						
30–39 years	28 (31.46%)	61 (68.54%)	Ref		Ref	
40–49 years	29 (25.44%)	85 (74.56%)	1.35	0.73, 2.49	1.55	1.02, 4.96
50–59 years	22 (30.14%)	51 (69.86%)	1.06	0.54, 2.09	1.18	0.63, 3.40
60+ years	10 (31.25%)	22 (68.75%)	1.01	0.43, 2.49	1.07	0.32, 3.72
Missing	0	0				
**Nationality**						
Non-German	13 (40.62%)	19 (59.38%)	Ref		Ref	
German	76 (27.54%)	200 (72.46%)	1.80	0.83, 3.80	1.58	0.96, 5.97
Missing	0	0				
**Study region**						
Mainz-Bingen (rural)	41 (24.12%)	129 (75.88%)	Ref		Ref	
Mainz (urban)	48 (34.78%)	90 (65.22%)	0.60	0.36, 0.98	0.63	0.30, 1.00
Missing	0	0				
**Education**						
Upper secondary or further^1^	36 (30.77%)	81 (69.23%)	Ref		Ref	
Lower secondary^2^	53 (27.75%)	138 (72.25%)	1.16	0.70, 1.91	1.01	0.75, 2.15
Missing	0	0				
**Employment**						
Employed	60 (27.91%)	155 (72.09%)	Ref		Ref	
Not employed^3^	22 (32.35%)	46 (67.65%)	0.81	0.45, 1.48	0.97	0.50, 1.83
Missing	7	18				
**Net household income**						
≤ 1500€/month	9 (13.43%)	58 (86.57%)	Ref		Ref	
> 1500€/month	58 (31.69%)	125 (68.31%)	0.33	0.15, 0.69	0.33	0.11, 0.92
Missing	22	36				
**Marital status**						
Married, divorced, widowed	69 (27.49%)	182 (72.51%)	Ref		Ref	
Single	17 (32.08%)	36 (67.92%)	0.80	0.43, 1.55	0.72	0.22, 1.12
Missing	3	1				
**Parity**						
0–2	64 (26.45%)	178 (73.55%)	Ref		Ref	
≥ 3	18 (46.15%)	21 (53.85%)	0.42	0.21, 0.84	0.32	0.10, 0.86
Missing	7	20				
**Smoking status**						
Never	34 (25.76%)	98 (74.24%)	Ref		Ref	
Ever	54 (31.03%)	120 (68.97%)	0.77	0.46, 1.27	0.76	0.32, 1.01
Missing	1	1				
**Oral contraceptive use**						
Never	17 (29.82%)	40 (70.18%)	Ref		Ref	
Ever	72 (28.80%)	178 (71.20%)	1.05	0.55, 1.95	0.90	0.28, 1.28
Missing	0	1				
**HRT**						
Never	74 (28.24%)	188 (71.76%)	Ref		Ref	
Ever	12 (31.58%)	26 (68.42%)	0.85	0.42, 1.83	0.96	0.29, 1.55
Missing	3	5				
**Health insurance**						
Statutory	54 (28.12%)	138 (71.88%)	Ref		Ref	
Private	9 (30.00%)	21 (70.00%)	0.91	0.40, 2.21	1.14	0.70, 4.62
Missing	26	60				
**Screening frequency**						
Regular^4^	70 (27.24%)	187 (72.76%)	Ref		Ref	
Irregular or never^5^	19 (40.43%)	28 (59.57%)	0.55	0.29, 1.06	0.82	0.30, 1.13
Missing	0	4				
**Screening result**						
ASC-US+ only	40 (47.62%)	44 (52.38%)	Ref		Ref	
hrHPV+ only	40 (22.47%)	138 (77.53%)	3.25^b^	1.91, 5.55	3.04^b^	1.49, 7.22
Both positive	9 (19.57%)	37 (80.43%)				

OR: odds ratio; CI: confidence interval; aOR: adjusted odds ratio; Ref: reference level; HRT: hormone replacement therapy; ASC-US+: Atypical squamous cells of undetermined significance or worse; hrHPV+: high-risk Human Papillomavirus positive; both positive: ASC-US+ and hrHPV positive

^1^ at least 12 years education

^2^ ≤ 10 years

^3^ includes other employment status such as parental leave, sick leave

^4^ every 1–2 years

^5^ every 3 years or less, irregular screening, rarely and no previous screening attendance

^a^ Adjusted for all covariates in the model

^b^ dichotomised to include hrHPV only and both co-test positive results (hrHPV and ASC-US+)

* Likelihood ratio

** Bootstrap resampled confidence intervals (n = 500)

### Reasons for non-attendance

**Fig. 3:**
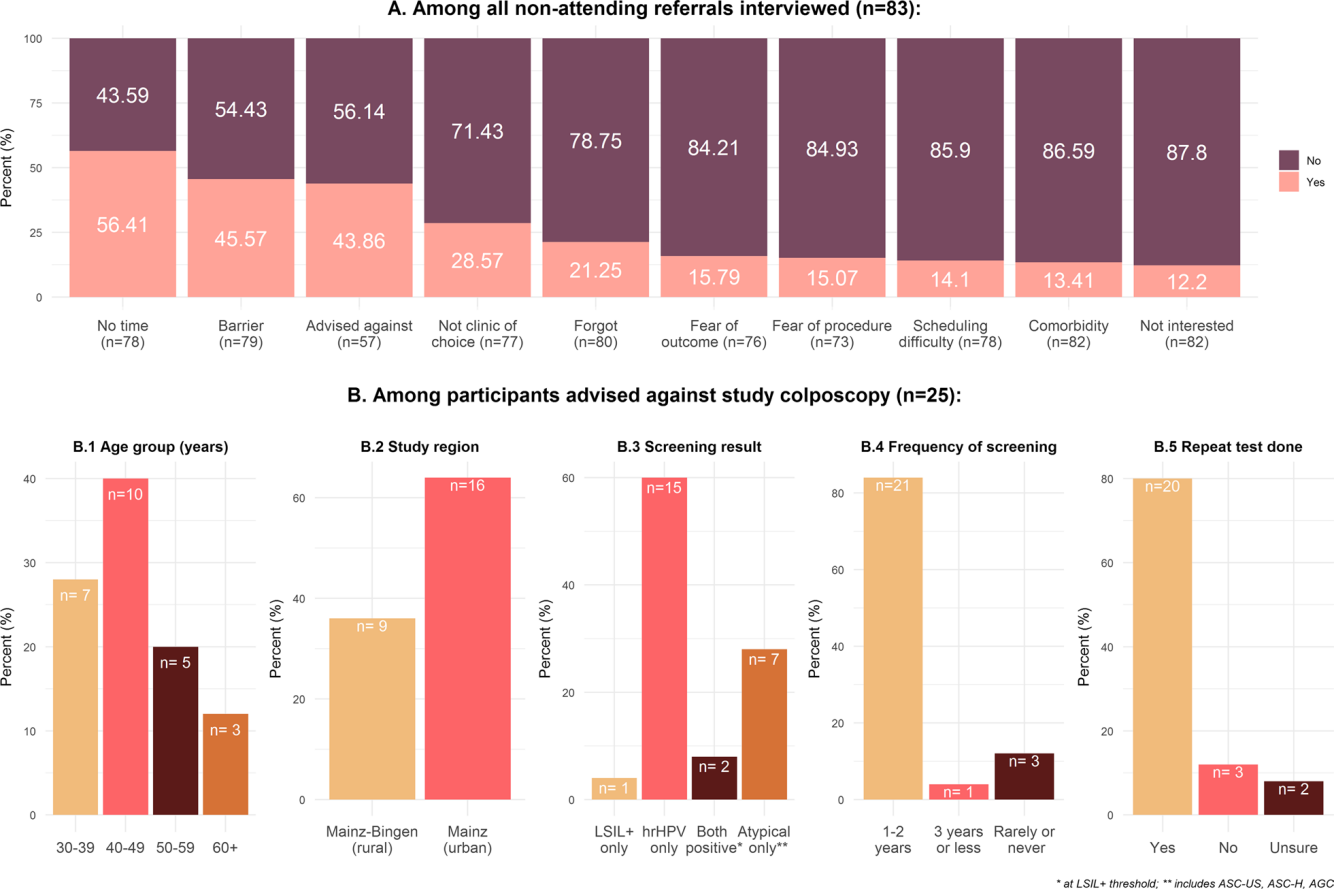
**A** Reasons for non-attendance over both rounds and **B** characteristics of the participants who were advised against attending the study colposcopy. ASC-US+: Atypical squamous cells of undetermined significance or worse; hrHPV: high-risk human Papillomavirus; LSIL+  low grade squamous intraepithelial lesion or worse

Overall, 83 respondents provided reasons on non-compliance (response rate R1: 68/77 (88.3%); R2: 18/23 (78.3%) (Fig. 3). Over half indicated lack of time (56%), almost half (48%) mentioned barriers such as long travel time, travel cost, childcare challenges and 29% cited lack of choice of colposcopy clinic (Fig. 3A). A fifth of the women reported to have forgotten the appointment, while 15–16% feared the procedure itself or the outcome of the examination (Fig. 3A).

Forty-four percent mentioned that their office-based gynaecologist who conducted screening advised against colposcopy at the study clinic (Fig. 3B). Thirty-six percent of these women resided in the urban region, 40% were aged 40–49 years, 8% had a positive co-test at the guideline threshold LSIL+ , 12% reported irregular or no screening history and 80% reported having a repeat test since the MARZY screening round (Figs. 3B.1–B.5). Among 9 co-test screen-positives (ASC-US+ and hrHPV positive) who did not attend at either round (Table 1), only one cited the advice of the screening gynaecologist as the main reason for non-attendance; the remainder reported other barriers or concerns (Additional file 1: Table S4).

### HPV: Perception, knowledge, communication and concerns

Among women who reported negative experiences during screening, 78% attended colposcopy compared to 87% of attendees who did not report a negative screening experience and 78% who reported moderate to high levels of negative reaction to their HPV result attended compared to 84% of attendees with little to no negative reaction (Table 2). Likelihood of attending colposcopy was lowered if screening was associated with a negative experience (OR = 0.49, 95% CI 0.21, 1.09) or reaction (OR = 0.64, 95% CI 0.27, 1.41), but not statistically significant. Approximately 79% of women reporting to have HPV knowledge attended compared to 82% with no HPV knowledge. Better levels of HPV knowledge were markedly lower among attendees (75%) than those reporting poor or no HPV knowledge who also attended colposcopy (82%). Level of understanding of the HPV result was not significantly associated with attendance (OR = 1.15, 95% CI 0.42, 2.82).

**Table 2:** HPV and screening-related factors of hrHPV positive women who underwent colposcopy versus hrHPV positive non-attendees

	Overall (n = 225)		Logistic regression model	
	Non-attendee (n = 49)	Attendee (n = 176)	Univariable	
	n (row %)	n (row %)	OR	95% CI
*Perception*				
**Negative screening experience**				
No	9 (13.04%)	60 (86.96%)	Ref	
Yes	26 (22.41%)	90 (77.59%)	0.49	0.21, 1.09
Missing	14	26		
**Level of negative reaction to HPV result**^a^				
Little to none	10 (15.87%)	53 (84.13%)	Ref	
Moderate to high	24 (21.82%)	86 (78.18%)	0.64	0.27, 1.41
Missing	15	37		
**Level of understanding regarding HPV result**				
Little to none	7 (22.58%)	24 (77.42%)	Ref	
Most or everything	27 (19.57%)	111 (80.43%)	1.15	0.42, 2.82
Missing	15	41		
*Knowledge*				
**HPV knowledge**				
No	17 (18.28%)	76 (81.72%)	Ref	
Yes	18 (21.18%)	67 (78.82%)	0.78	0.37, 1.62
Missing	14	33		
**Level of HPV knowledge**				
Poor to none	5 (14.71%)	29 (85.29%)	Ref	
Moderate to good	13 (25.00%)	39 (75.00%)	0.64	0.20, 1.84
Missing	31	108		
**Any HPV knowledge prior to the study**				
No	16 (17.78%)	74 (82.22%)	Ref	
Yes	17 (19.32%)	71 (80.68%)	0.84	0.39, 1.78
Missing	16	31		
*Communication*				
**Of HPV result by gynaecologist**				
No	8 (24.24%)	25 (75.76%)	Ref	
Yes	4 (15.38%)	22 (84.62%)	1.34	0.39, 5.03
Missing	37	129		
**Comprehensive explanation of HPV result by gynaecologist**^b^				
1 area or less	27 (20.00%)	108 (80.00%)	Ref	
At least 2 areas	7 (16.67%)	35 (83.33%)	1.06	0.46, 2.70
Missing	15	33		
**Trust in gynaecologist**				
No	3 (15.00%)	17 (85.00%)	Ref	
Yes	23 (19.01%)	98 (80.99%)	0.71	0.16, 2.34
Unsure*	8 (27.59%)	21 (72.41%)		
Missing	15	40		
**Discussed HPV result with friend or family**				
No	16 (26.23%)	45 (73.77%)	Ref	
Yes	18 (16.22%)	93 (83.78%)	1.72	0.80, 3.67
Missing	15	38		
*Concerns*				
**About cancer**				
No	8 (18.60%)	35 (81.40%)	Ref	
Yes	27 (19.29%)	113 (80.71%)	0.91	0.36, 2.11
Missing	14	28		
**About infertility**				
No	27 (18.88%)	116 (81.12%)	Ref	
Yes	8 (24.24%)	25 (75.76%)	0.76	0.32, 1.96
Missing	14	35		
**Of infecting partner**				
No	25 (20.83%)	95 (79.17%)	Ref	
Yes	8 (15.38%)	44 (84.62%)	1.52	0.66, 3.84
Missing	16	37		
**About impact on sexual intercourse**				
No	26 (19.85%)	105 (80.15%)	Ref	
Yes	8 (16.67%)	40 (83.33%)	1.30	0.56, 3.27
Missing	15	31		

OR: Odds Ratio; CI: Confidence Interval; HPV: Human Papillomavirus; hrHPV: high-risk human Papillomavirus

^a^ at least one of the following: anxiety, insecurity, nervousness, incomprehension, powerlessness

^b^ areas include: dedicated time for explaining result, background information on HPV, answered questions or concerns from patient

* not included in logistic regression

For communication, 85% of attendees reported direct communication of the HPV result by their gynaecologist compared to 76% of colposcopy attendees who were not directly informed by the gynaecologist. Direct communication increased the likelihood of attending but was not statistically significant (OR = 1.34, 95% CI 0.39, 5.03). Higher proportions of attendees also reported comprehensive counselling (83% vs. 80%), and discussed their result with a friend or family member (84% vs. 74%) than those who did not report these discussions. Eighty-five percent of women who reported lack of trust in their gynaecologist went to colposcopy compared to 81% who reported trust. Approximately 77% of all hrHPV positive women who responded in Q3 were concerned about cancer.

### Longitudinal outcomes

At baseline R1, 77 referrals to colposcopy at R1 did not attend. Of these, 44 were lost to follow-up and not subsequently screened at R2. Respectively, baseline data and retrospective documentation of outcomes among these women indicated that 25 women (57%) were at least hrHPV positive (hrHPV positive only or both ASC-US+ and hrHPV positive) and a total of 4 women were scheduled to later undergo hysterectomies outside of the study (Additional file 1: Table S5). Three of the 4 women who underwent hysterectomies had a positive screening result within routine screening after R1 of MARZY.

Among the 33 referrals who did not attend colposcopy at R1 and were re-screened at R2, the majority (92%) were screened routinely between study rounds with negative screening results (Table 3). Only 2 non-attendees from R1 were screen-positive upon routine screening after study baseline. At R2, 4 women (12%) were hrHPV positive only, 2 (6%) were co-test positive to both cytology and hrHPV, while 27 (82%) were screen-negative. Among the 6 women referred again to colposcopy at R2, 5 (83%) did not attend, despite all 5 having a hrHPV positive result detected at R2 screening. Two of these women also had a concurrent cytological abnormality and via retrospective data linkage, it was found that they later underwent hysterectomies due to severe cervical intraepithelial neoplasia or worse (CIN3+ ; Additional file 1: Table S6).

**Table 3:** Longitudinal outcomes of baseline round (R1) referred women who were also screened at the MARZY follow-up round (R2)

Outcome	Non-attendee at R1 (n = 33)	Attendee at R1 (n = 109)
*Between MARZY study rounds*		
**Screening result**		
Positive	2 (7.69%)	19 (21.35%)
Negative (attended routine screening)	24 (92.31%)	66 (74.16%)
Did not undergo any screening since R1	0 (0.00%)	4 (4.49%)
Missing	7	20
Total	33	109
*At MARZY study R2*		
**Screening test result**		
ASC-US+ only	0 (0.00%)	3 (2.75%)
hrHPV+ only	4 (12.12%)	8 (7.34%)
Both positive	2 (6.06%)	4 (3.67%)
Negative	27 (81.82%)	94 (86.24%)
Total	33	109
**Colposcopy referred and attendance status**		
No attendance	5 (83.33%)	6 (40.00%)
Attended	1 (16.67%)	9 (60.00%)
Not applicable (screen-negative)	27	94
Total	33	109

ASC-US+: Atypical squamous cells of undetermined significance or worse; hrHPV+: high-risk human Papillomavirus positive; both positive: ASC-US+ and hrHPV positive

Characteristics of the non-attendees who presented again at R2 show that 67% were women aged 50 years and above and 61% resided in an urban area (Additional file 1: Table S7). Twenty-one percent had 3 or more children and 28% did not attend screening regularly. Sixty-four percent of non-attendees from R1 reported no time as a reason for non-attendance at R1 and 50% reported a barrier. Among the 5 referrals who did not attend colposcopy at either R1 or R2, common reasons were lack of time, concerns and obstacles to arranging the appointment (Additional file 1: Table S6).

## Discussion

In a population-based cohort study with both cytological and HPV testing (co-testing), the overall proportion of colposcopy non-attendance in screen-positive women was 29%. In referrals with ASC-US+ and hrHPV positive results, 20% did not attend despite active recall efforts. Attendance was associated with having a positive HPV status. Lack of time, barriers including childcare arrangements, travel time as well as lack of clinic choice and the advice given by the gynaecologist who conducted screening were cited as major reasons for non-attendance.

We observed higher non-attendance than in Europe (6–10%) [13, 16, 33]. In North America where CCS is offered opportunistically, non-attendance was observed in 28% of screened women [17], and up to 44% in underserved populations [34]. Low proportions of non-attendance appear to stem from organised screening contexts with active referral to colposcopy. This most likely explains the higher non-attendance rate observed in our study, since screening in Germany until 2020 was opportunistic. Historically, expert colposcopy was also not routinely performed, partly due to the annual screening interval, lack of certified dysplasia centres [35] and gynaecologists conducting repeat smears instead. This is evident in the high proportion of women in our study who were advised by their gynaecologist not to attend colposcopy and instead underwent repeat screening. Additionally, the guideline in effect at the time, when HPV screening was not offered, did not include recommendations for positive HPV or co-test results. The discrepancy between guideline and study protocol could explain this advice.

High non-adherence rates also arise from the lack of a screening registry to systematically contact non-attendees and lack of personnel to conduct recalls in non-organised programmes [36]. Randomised trials and community programs have demonstrated written reminders, preclinic calls and communication with patients significantly increase adherence to follow-up care [20, 23]. In our study, we were able to motivate a third of non-attending women to attend colposcopy by active call-recall. However, this may pose logistical challenges as the communication of results and referral is the responsibility of the screening physician, both in the previous and current screening program in Germany [5]. Management gaps between screening physicians and dysplasia centres where colposcopies are conducted also exist [35]. Enhanced patient communication conducted by clinic staff, streamlined management between gynaecological care providers and integration within a standardised call-recall system need to be introduced to reduce anxiety and improve attendance. Similar to other countries with organised screening, a programme target of less than 15% non-attendance should also be set [12].

Almost half (48%) of referrals with cytological abnormalities did not attend colposcopy, probably due to the annual screening interval. Congruent to a recent pilot study [24], a positive hrHPV result significantly increased attendance in our study by three times. We screened participants with HPV testing in addition to cytology, which at the time was not part of routine CCS in Germany. As the majority of hrHPV referrals reported concerns about cancer in our study, additional HPV testing may have caused anxiety or concern [37], which might have led to better attendance. However, in a randomised trial to reduce anxiety by educating participants on HPV before colposcopy, knowledge significantly increased but anxiety did not decrease [38]. Balanced risk communication must be addressed in a programme that offers HPV screening, and could be differential for subgroups such as younger and older women [39]. Furthermore, attendance rates could be improved if engaging information on colposcopy and particular attention for the emotional experience are provided [25, 26]. This is important since concerns and barriers were noted as reasons for non-attendance in a small group of women that did not attend colposcopy, despite being referred in both rounds.

Women with several children were less likely to attend colposcopy. Indeed, the major reasons cited for non-attendance were lack of time and barriers including lack of childcare arrangements, transport times and general lack of clinic choice (hospitals only). Additionally, our active recall efforts may not have mitigated such barriers, rather that it was more effective among women with hesitations. Moreover, we observed better attendance among women who were communicated their positive hrHPV result by the screening gynaecologist, in alignment with previous findings [23]. In a meta-analysis, even after HPV self-sampling kits are offered as a method to address barriers, follow-up non-adherence remains around 19% [40]. These observations underscore the necessity to diversify follow-up alternatives (self-sampling) and the importance of an established relationship including trust between the patient and physician. As recall appears largely to be left to the responsibility of the provider [5], encouraging information packs, educational support for screening physicians in counselling patients backed by a systematic screening registry for call-recall should be provided [12].

### Limitations

We defined non-attendees as screen-positive to either cytology or HPV testing, rather than both cytology and HPV test positive. This may overestimate non-attendance as many who are screen-positive to one test only would normally undergo repeat Pap smear 3, 6 or 12 months later according to the guidelines in effect at the time in Germany [29]. However after restricting non-attendance to positive co-test results (ASC-US+ or LSIL+ and hrHPV positive), we found similar attendance rates. The sample size may have also restricted our analyses, particularly for the HPV-related items in Q3. However, only 18% of hrHPV positive cases were Q3 non-respondents. Additional assessment between Q3 respondents and non-respondents revealed some differences in nationality and socioeconomic status (Additional file 1: Table S8). These differences highlight potential external validity limitations of our results to un(der)screened women. Some non-attendees whom were unreachable may have sought colposcopy elsewhere, but the numbers are small.

## Conclusion

Our population-based screening study offers important insight into colposcopy non-attendance, particularly as HPV testing is being integrated into screening in many countries. We quantitatively and qualitatively described the major reasons for non-attendance, which is important to maximise screening effectiveness. A considerable proportion of women did not attend colposcopy after abnormal screening results, and this persisted even in some women who were referred twice. Certain subgroups of women could be targeted by personalised measures within a failsafe recall system, especially since HPV testing is new. Continued educational support of screening gynaecologists should also be integrated. An optimised screening management continuum can reduce loss to follow up, minimise preventable CC diagnoses and improve the overall effectiveness of cancer screening.

## Supplementary Information

**Additional file 1**. The following file contains further analyses and insight (tables S1–S8 and figure S1) to complement the main analyses of our study

## Data Availability

The datasets used and/or analysed during the current study are available from the corresponding author on reasonable request.

## Abbreviations

ASC-US: Atypical squamous cells of undetermined significance
CC: Cervical cancer
CCS: Cervical cancer screening
CIN: Cervical intraepithelial neoplasia
HC2: Hybrid Capture®2
HPV: Human papillomavirus
hrHPV: High-risk HPV
LSIL: Low-grade squamous intraepithelial lesion
