# Supplementary material for: Colposcopy non-attendance following an abnormal cervical cancer screening result: a prospective population-based cohort study
Source: BMC Womens Health. 2022 Jul 9;22:285. doi: 10.1186/s12905-022-01851-6 (PMC9270801; doi:10.1186/s12905-022-01851-6)
Supplement: Supplementary file 1 — Additional file 1. The following file contains further analyses and insight (tables S1–S8 and figure S1) to complement the main analyses of our study [file 12905_2022_1851_MOESM1_ESM.docx]

# Additional file 1

The following file contains further analyses and insight (tables S1-S8 and figure S1) to complement the main analyses of our study.

**Table S1.** Sociodemographic and lifestyle factors of participants at round 1 and round 2

|  | **Baseline R1 (n=222)** | | **Follow-up R2 (n=107*)** | |
| --- | --- | --- | --- | --- |
|  | **Non-attendee (n=77)** | **Attendee (n=145)** | **Non-attendee (n=23)** | **Attendee (n=84)** |
| **Mean age (standard deviation)** | 45.71 (10.23) | 44.98 (9.29) | 45.87 (10.34) | 47.06 (8.62) |
|  | **n (row %)** | **n (row %)** | **n (row %)** | **n (row %)** |
| **Age group** |  |  |  |  |
| 30-39 years | 24 (34.78%) | 45 (65.22%) | 9 (31.03%) | 20 (68.97%) |
| 40-49 years | 23 (28.40%) | 58 (71.60%) | 7 (18.42%) | 31 (81.58%) |
| 50-59 years | 22 (44.00%) | 28 (56.00%) | 4 (14.29%) | 24 (85.71%) |
| 60+ years | 8 (36.36%) | 14 (63.64%) | 3 (25.00%) | 9 (75.00%) |
| Missing | 0 | 0 | 0 | 0 |
| **Nationality** |  |  |  |  |
| Non-German | 9 (39.13%) | 14 (60.87%) | 5 (45.45%) | 6 (54.55%) |
| German | 68 (34.17%) | 131 (65.83%) | 18 (18.75%) | 78 (81.25%) |
| Missing | 0 | 0 | 0 | 0 |
| **Study region** |  |  |  |  |
| Mainz-Bingen (rural) | 37 (28.68%) | 92 (71.32%) | 8 (15.09%) | 45 (84.91%) |
| Mainz (urban) | 40 (43.01%) | 53 (56.99%) | 15 (27.78%) | 39 (72.22%) |
| Missing | 0 | 0 | 0 | 0 |
| **Education** |  |  |  |  |
| 9 years or less | 24 (30.77%) | 54 (69.23%) | 6 (19.35%) | 25 (80.65%) |
| 10 years | 23 (39.66%) | 35 (60.34%) | 4 (11.43%) | 31 (88.57%) |
| 12-13 years | 30 (34.88%) | 56 (65.12%) | 13 (31.71%) | 28 (68.29%) |
| Missing | 0 | 0 | 0 | 0 |
| **Employment** |  |  |  |  |
| Employed | 53 (35.10%) | 98 (64.90%) | 13 (17.11%) | 63 (82.89%) |
| Not employed | 14 (29.79%) | 33 (70.21%) | 7 (41.18%) | 10 (58.82%) |
| Other | 8 (38.10%) | 13 (61.90%) | 2 (16.67%) | 10 (83.33%) |
| Missing | 2 | 1 | 1 | 1 |
| **Net household income (€/month)** |  |  |  |  |
| ≤1500 | 8 (17.02%) | 39 (82.98%) | 5 (18.52%) | 22 (81.48%) |
| >1500 | 48 (37.21%) | 81 (62.79%) | 14 (21.88%) | 50 (78.12%) |
| Missing | 21 | 25 | 4 | 12 |
| **Marital status** |  |  |  |  |
| Single | 15 (37.50%) | 25 (62.50%) | 6 (31.58%) | 13 (68.42%) |
| Married, divorced, widowed | 59 (33.15%) | 119 (66.85%) | 17 (19.32%) | 71 (80.68%) |
| Missing | 3 | 1 | 0 | 0 |
| **Parity** |  |  |  |  |
| 0-2 | 56 (32.94%) | 114 (67.06%) | 16 (18.18%) | 72 (81.82%) |
| ≥3 | 15 (46.88%) | 17 (53.12%) | 5 (45.45%) | 6 (54.55%) |
| Missing | 6 | 14 | 2 | 6 |
| **Smoking status** |  |  |  |  |
| Never | 29 (30.85%) | 65 (69.15%) | 9 (19.15%) | 38 (80.85%) |
| Ever | 47 (37.30%) | 79 (62.70%) | 14 (23.33%) | 46 (76.67%) |
| Missing | 1 | 1 | 0 | 0 |
| **Oral contraceptive use** |  |  |  |  |
| Never | 13 (29.55%) | 31 (70.45%) | 5 (29.41%) | 12 (70.59%) |
| Ever | 64 (36.16%) | 113 (63.84%) | 18 (20.00%) | 72 (80.00%) |
| Missing | 0 | 1 | 0 | 0 |
| **HRT** |  |  |  |  |
| Never | 62 (33.70%) | 122 (66.30%) | 20 (21.05%) | 75 (78.95%) |
| Ever | 12 (40.00%) | 18 (60.00%) | 2 (18.18%) | 9 (81.82%) |
| Missing | 3 | 5 | 1 | 0 |
| **Health insurance** |  |  |  |  |
| Statutory | 49 (33.56%) | 97 (66.44%) | 14 (22.95%) | 47 (77.05%) |
| Private | 7 (33.33%) | 14 (66.67%) | 3 (30.00%) | 7 (70.00%) |
| Missing | 21 | 34 | 6 | 30 |
| **Screening frequency** |  |  |  |  |
| Regular^a^ | 62 (33.33%) | 124 (66.67%) | 17 (18.89%) | 73 (81.11%) |
| Irregular or never^b^ | 15 (45.45%) | 18 (54.55%) | 6 (37.50%) | 10 (62.50%) |
| Missing | 0 | 3 | 0 | 1 |
| **Positive screening result between rounds** |  |  |  |  |
| Yes | 27 (28.72%) | 67 (71.28%) | 12 (26.67%) | 33 (73.33%) |
| No | 50 (39.06%) | 78 (60.94%) | 11 (17.74%) | 51 (82.26%) |

* includes 21 participants who were also referred to colposcopy at baseline R1.
^a^ every 1-2 years
^b^ every 3 years or less, irregular screening, rarely and no previous screening attendance
HRT: hormone replacement therapy

**Table S2.** Age and screening result among 21 women referred to colposcopy twice over the MARZY study

|  | **Baseline R1** | | | **Follow-up R2** | | |
| --- | --- | --- | --- | --- | --- | --- |
|  | **Age (years)** | **Screening result** | **Colposcopy attendance status** | **Age (years)** | **Screening result** | **Colposcopy attendance status** |
| 1 | 54 | hrHPV+ only | Attendee | 57 | hrHPV+ only | Attendee |
| 2 | 37 | hrHPV+ only | Attendee | 40 | hrHPV+ only | Attendee |
| 3 | 42 | hrHPV+ only | Attendee | 45 | hrHPV+ only | Attendee |
| 4 | 56 | hrHPV+ only | Non-attendee | 59 | hrHPV+ only | Non-attendee |
| 5 | 37 | Both positive | Attendee | 40 | hrHPV+ only | Attendee |
| 6 | 39 | hrHPV+ only | Attendee | 42 | Both positive | Attendee |
| 7 | 65 | Both positive | Attendee | 67 | hrHPV+ only | Attendee |
| 8 | 58 | hrHPV+ only | Non-attendee | 60 | hrHPV+ only | Non-attendee |
| 9 | 37 | hrHPV+ only | Attendee | 40 | Both positive | Non-attendee |
| 10 | 41 | Both positive | Attendee | 43 | Both positive | Attendee |
| 11 | 59 | ASC-US+ only | Attendee | 61 | ASC-US+ only | Non-attendee |
| 12 | 39 | Both positive | Attendee | 43 | hrHPV+ only | Non-attendee |
| 13 | 39 | ASC-US+ only | Non-attendee | 43 | hrHPV+ only | Attendee |
| 14 | 35 | Both positive | Attendee | 39 | hrHPV+ only | Non-attendee |
| 15 | 32 | hrHPV+ only | Attendee | 35 | hrHPV+ only | Non-attendee |
| 16 | 42 | hrHPV+ only | Attendee | 45 | Both positive | Attendee |
| 17 | 40 | hrHPV+ only | Non-attendee | 43 | Both positive | Non-attendee |
| 18 | 36 | Both positive | Attendee | 39 | ASC-US+ only | Non-attendee |
| 19 | 43 | ASC-US+ only | Attendee | 45 | ASC-US+ only | Attendee |
| 20 | 55 | hrHPV+ only | Non-attendee | 57 | hrHPV+ only | Non-attendee |
| 21 | 61 | Both positive | Non-attendee | 63 | Both positive | Non-attendee |

ASC-US+: Atypical squamous cells of undetermined significance or worse; hrHPV+: high-risk human Papillomavirus positive; both positive: ASC-US+ and hrHPV positive

**Table S3.** Cytology subgroup result and colposcopy attendance status among all referrals over both rounds

| **Cytology result Munich Nomenclature II (Bethesda Classification equivalent)** | **Non-attendee  (n=40)** | **Attendee  (n=44)** |
| --- | --- | --- |
| Pap I/II (NILM) | NA | NA |
| Pap IIw/IIk (ASC-US) | 31 (50.00%) | 31 (50.00%) |
| Pap III (ASC-H, AGC) | 3 (33.33%) | 6 (66.67%) |
| Pap IIID (LSIL, HSIL) | 6 (54.55%) | 5 (45.45%) |
| Pap IV (HSIL, AIS; [with features suspicious for invasion]) | 0 (0.00%) | 2 (100.00%) |
| Pap V (SCC, AC) | 0 | 0 |

NA: Not applicable; NILM: Negative for intraepithelial lesion/ or malignancy; ASC-US: Atypical squamous cells of undetermined significance; ASC-H: Atypical squamous cells of undetermined significance, cannot exclude HSIL; AGC: Atypical glandular cells; LSIL: Low grade squamous intraepithelial lesion; HSIL: High grade squamous intraepithelial lesion; SCC: Squamous cell carcinoma; AC: Adenocarcinoma


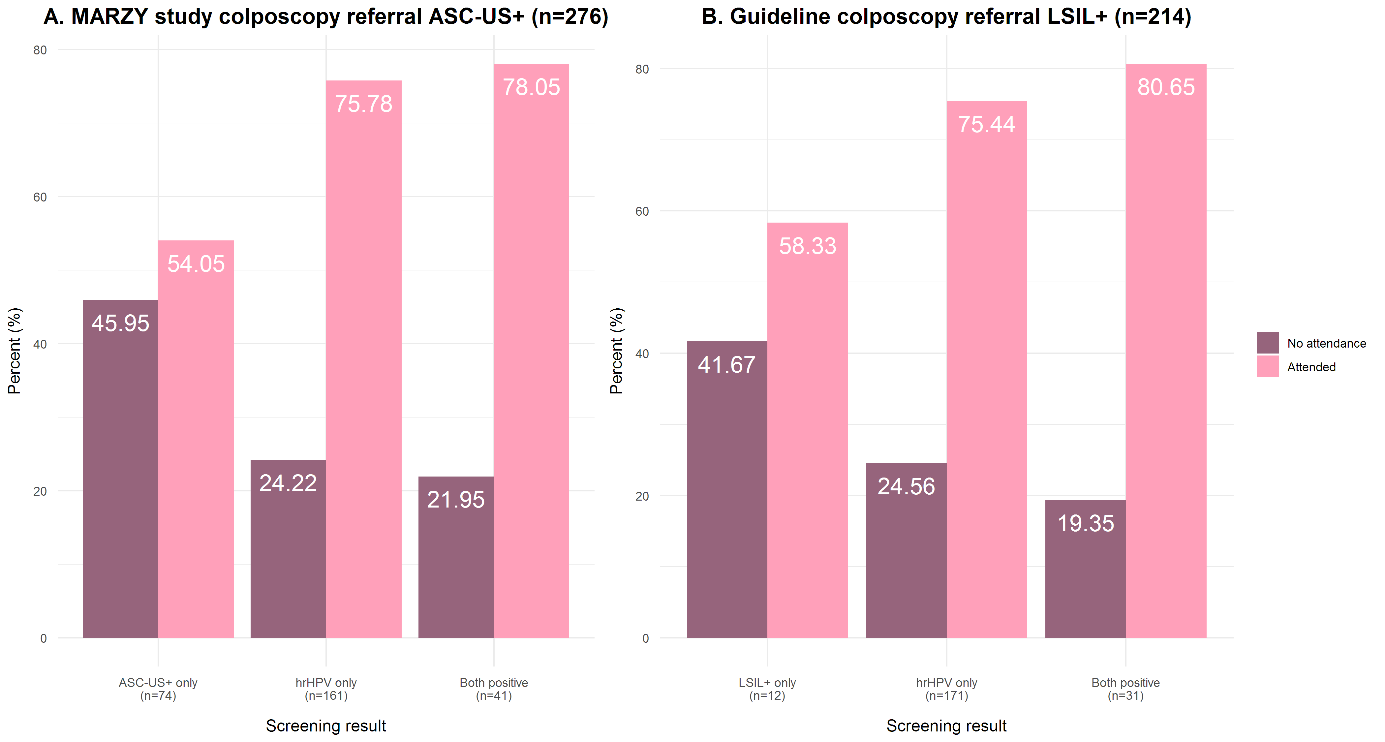


**Figure S1.** Proportion of referrals who attended or did not attend colposcopy excluding any positive cytology cases detected within routine Pap screening between study rounds

ASC-US+: Atypical squamous cells of undetermined significance or worse; hrHPV: high-risk human Papillomavirus; LSIL+: low grade squamous intraepithelial lesion or worse

**Table S4.** Characteristics and reasons for non-attendance among 9 colposcopy non-attendees who were co-test positive (ASC-US+ and hrHPV+)

| **Non-attendee** | **Age (years)** | **Residency** | **Frequency of screening attendance** | **Reason(s) for non-attendance** |
| --- | --- | --- | --- | --- |
| **1** | 50 | Mainz-Bingen (rural) | Every 1-2 years | No time |
| **2** | 34 | Mainz (urban) | Every 1-2 years | *NA - Could not be reached* |
| **3** | 41 | Mainz-Bingen (rural) | Every 3 years or more | Comorbidity |
| **4** | 38 | Mainz-Bingen (rural) | Every 1-2 years | Physician advised against, fear of the result |
| **5** | 50 | Mainz-Bingen (rural) | Every 1-2 years | No time, barrier, lack of clinic choice, difficulty with scheduling appointment |
| **6** | 42 | Mainz-Bingen (rural) | Every 1-2 years | *NA - Could not be reached* |
| **7** | 58 | Mainz (urban) | Every 3 years or more | No time, difficulty with scheduling, not interested, had a repeat smear done |
| **8** | 61 | Mainz (urban) | Rarely or never | Barrier, forgot, fear of the result |
| **9** | 35 | Mainz (urban) | Every 1-2 years | *NA - Could not be reached* |

ASC-US+: Atypical squamous cells of undetermined significance or worse; hrHPV+: high-risk human Papillomavirus positive

**Table S5.** Screening results and outcomes of non-attendees referred at baseline round (R1) who were lost to follow-up at the subsequent MARZY screening round (R2)

| **Non attendee at R1** | **Screening result at R1** | **Documented treatment after R1** | **Screening result between MARZY rounds** |
| --- | --- | --- | --- |
| 1 | ASC-US+ only | - | - |
| 2 | hrHPV+ only | - | - |
| 3 | hrHPV+ only | - | - |
| 4 | hrHPV+ only | - | - |
| 5 | hrHPV+ only | - | - |
| 6 | ASC-US+ only | - | - |
| 7 | ASC-US+ only | - | - |
| 8 | ASC-US+ only | - | - |
| 9 | ASC-US+ only | - | - |
| 10 | hrHPV+ only | - | - |
| 11 | hrHPV+ only | - | - |
| 12 | hrHPV+ only | - | - |
| 13 | hrHPV+ only | Hysterectomy | - |
| 14 | hrHPV+ only | - | - |
| 15 | hrHPV+ only | - | - |
| 16 | hrHPV+ only | - | - |
| 17 | ASC-US+ only | - | - |
| 18 | hrHPV+ only | - | - |
| 19 | ASC-US+ only | - | - |
| 20 | ASC-US+ only | - | - |
| 21 | ASC-US+ only | - | - |
| 22 | hrHPV+ only | - | - |
| 23 | hrHPV+ only | - | - |
| 24 | both positive | Hysterectomy | Positive |
| 25 | ASC-US+ only | - | - |
| 26 | hrHPV+ only | - | - |
| 27 | hrHPV+ only | - | - |
| 28 | both positive | - | - |
| 29 | ASC-US+ only | - | - |
| 30 | ASC-US+ only | Hysterectomy | Positive |
| 31 | ASC-US+ only | - | - |
| 32 | hrHPV+ only | - | - |
| 33 | hrHPV+ only | - | - |
| 34 | ASC-US+ only | - | - |
| 35 | hrHPV+ only | - | - |
| 36 | hrHPV+ only | - | - |
| 37 | ASC-US+ only | - | - |
| 38 | ASC-US+ only | - | - |
| 39 | hrHPV+ only | - | - |
| 40 | ASC-US+ only | - | - |
| 41 | ASC-US+ only | - | - |
| 42 | both positive | Hysterectomy | Positive |
| 43 | both positive | - | - |
| 44 | ASC-US+ only | - | - |

- missing data
ASC-US+: Atypical squamous cells of undetermined significance or worse; hrHPV+: high-risk human Papillomavirus positive; both positive: ASC-US+ and hrHPV positive

**Table S6.** Outcomes and reasons among 5 non-attendees who were referred at both MARZY screening rounds but did not go to colposcopy at either round

| **Non-attendee** | **Screening result at R1** | **Screening result between rounds** | **Screening result at R2** | **Documented histopathology result after R2** | **Documented treatment after R2** | **Reason(s) for non-attendance** |
| --- | --- | --- | --- | --- | --- | --- |
| 1 | hrHPV+ only | - | hrHPV+ only | - | - | No time, lack of clinic choice, forgot, fear of the procedure |
| 2 | hrHPV+ only | - | hrHPV+ only | - | - | No time, physician advised against, comorbidity |
| 3 | hrHPV+ only | - | both positive | CIN3 | Hysterectomy | *NA - Could not be reached* |
| 4 | hrHPV+ only | - | hrHPV+ only | - | - | No time, difficulty with scheduling appointment |
| 5 | both positive | Positive | both positive | SCC | Hysterectomy | *NA - Could not be reached* |

- missing data
high-risk human Papillomavirus positive; both positive: Atypical squamous cells of undetermined significance or worse (ASC-US+) and hrHPV positive; CIN3: severe cervical intraepithelial neoplasia grade; SCC: squamous cell carcinoma

**Table S7.** Overview of non-attendees referred at baseline round (R1) who were also screened at follow-up round (R2)

|  | **Non-attendee at R1 (n=33)** | **Attendee at R1  (n=109)** |
| --- | --- | --- |
| **Characteristics** |  |  |
| **Age group** |  |  |
| 30-39 years | 7 (21.21%) | 20 (18.35%) |
| 40-49 years | 4 (12.12%) | 48 (44.04%) |
| 50-59 years | 14 (42.42%) | 25 (22.94%) |
| 60+ years | 8 (24.24%) | 16 (14.68%) |
| **Nationality** |  |  |
| Non-German | 2 (6.06%) | 8 (7.34%) |
| German | 31 (93.94%) | 101 (92.66%) |
| **Study region** |  |  |
| Mainz-Bingen (rural) | 13 (39.39%) | 67 (61.47%) |
| Mainz (urban) | 20 (60.61%) | 42 (38.53%) |
| **Education** |  |  |
| Upper secondary or further^1^ | 20 (62.50%) | 58 (54.21%) |
| Lower secondary^2^ | 12 (37.50%) | 49 (45.79%) |
| Missing | 1 | 2 |
| **Smoking status** |  |  |
| Never | 13 (39.39%) | 44 (40.37%) |
| Ever | 20 (60.61%) | 65 (59.63%) |
| Missing |  |  |
| **Parity** |  |  |
| 0-2 | 23 (79.31%) | 88 (86.27%) |
| ≥3 | 6 (20.69%) | 14 (13.73%) |
| Missing | 4 | 7 |
| **Screening frequency** |  |  |
| Regular^3^ | 27 (81.82%) | 96 (88.89%) |
| Irregularly^4^ | 1 (3.03%) | 5 (4.63%) |
| Rarely or never | 5 (15.15%) | 7 (6.48%) |
| Missing | 0 | 1 |
| **Reasons for non-attendance at R1** |  |  |
| **No time** |  |  |
| No | 10 (35.71%) |  |
| Yes | 18 (64.29%) |  |
| Missing | 5 |  |
| **Gynaecologist advised against** |  |  |
| No | 12 (57.14%) |  |
| Yes | 9 (42.86%) |  |
| Missing | 12 |  |
| **Barrier** |  |  |
| No | 14 (50.00%) |  |
| Yes | 14 (50.00%) |  |
| Missing | 5 |  |
| **Did not want to go to study centre** |  |  |
| No | 19 (67.86%) |  |
| Yes | 9 (32.14%) |  |
| Missing | 5 |  |
| **Forgot** |  |  |
| No | 23 (82.14%) |  |
| Yes | 5 (17.86%) |  |
| Missing | 5 |  |
| **Appointment difficult to obtain** |  |  |
| No | 25 (89.29%) |  |
| Yes | 3 (10.71%) |  |
| Missing | 5 |  |
| **Fear of the procedure** |  |  |
| No | 27 (100.00%) |  |
| Yes | 0 (0.00%) |  |
| Missing | 6 |  |
| **Fear of the resulting outcome** |  |  |
| No | 22 (81.48%) |  |
| Yes | 5 (18.52%) |  |
| Missing | 6 |  |
| **Comorbidity or undergoing other health procedure** |  |  |
| No | 25 (89.29%) |  |
| Yes | 3 (10.71%) |  |
| Missing | 5 |  |
| **Not interested** |  |  |
| No | 25 (89.29%) |  |
| Yes | 3 (10.71%) |  |
| Missing | 5 |  |

^1^ at least 12 years education
^2^ ≤10 years
^3^ every 1-2 years
^4^ every 3 years or more

**Table S8.** Comparison of respondents and non-respondents who were hrHPV positive and surveyed (Q3)

|  | **Q3 (HPV-related)** | |
| --- | --- | --- |
|  | **Non-respondents (N=40)** | **Respondents (N=185)** |
| **Age group** |  |  |
| 30-39 years | 14 (35.00%) | 60 (32.43%) |
| 40-49 years | 14 (35.00%) | 61 (32.97%) |
| 50-59 years | 6 (15.00%) | 45 (24.32%) |
| 60+ years | 6 (15.00%) | 19 (10.27%) |
| **Nationality** |  |  |
| Non-German | 6 (15.00%) | 14 (7.57%) |
| German | 34 (85.00%) | 171 (92.43%) |
| **Study region** |  |  |
| Mainz-Bingen (rural) | 19 (47.50%) | 109 (58.92%) |
| Mainz (urban) | 21 (52.50%) | 76 (41.08%) |
| **Education** |  |  |
| Upper secondary or further^1^ | 26 (65.00%) | 112 (60.54%) |
| Lower secondary^2^ | 14 (35.00%) | 73 (39.46%) |
| **Employment** |  |  |
| Employed | 25 (69.44%) | 134 (77.46%) |
| Not employed^3^ | 11 (30.56%) | 39 (22.54%) |
| Missing | 4 | 12 |
| **Net household income** |  |  |
| ≤1500€/month | 12 (54.55%) | 46 (28.22%) |
| >1500€/month | 10 (45.45%) | 117 (71.78%) |
| Missing | 18 | 22 |
| **Marital status** |  |  |
| Married, divorced, widowed | 8 (21.05%) | 33 (17.84%) |
| Single | 30 (78.95%) | 152 (82.16%) |
| Missing | 2 | 0 |
| **Parity** |  |  |
| 0-2 | 27 (84.38%) | 148 (87.57%) |
| ≥3 | 5 (15.62%) | 21 (12.43%) |
| Missing | 8 | 16 |
| **Smoking status** |  |  |
| Never | 17 (42.50%) | 76 (41.53%) |
| Ever | 23 (57.50%) | 107 (58.47%) |
| Missing | 0 | 2 |
| **Oral contraceptive use** |  |  |
| Never | 5 (12.50%) | 34 (18.48%) |
| Ever | 35 (87.50%) | 150 (81.52%) |
| Missing | 0 | 1 |
| **HRT** |  |  |
| Never | 33 (86.84%) | 156 (86.19%) |
| Ever | 5 (13.16%) | 25 (13.81%) |
| Missing | 2 | 4 |
| **Health insurance** |  |  |
| Statutory | 28 (90.32%) | 117 (87.31%) |
| Private | 3 (9.68%) | 17 (12.69%) |
| Missing | 9 | 51 |
| **Screening frequency** |  |  |
| Regular^4^ | 33 (82.50%) | 161 (88.46%) |
| Irregular or never^5^ | 7 (17.50%) | 21 (11.54%) |
| Missing | 0 | 3 |

^1^ at least 12 years education
^2^ ≤10 years
^3^ includes other employment status
^4^ every 1-2 years
^5^ every 3 years or less, irregular screening, rarely and no previous screening attendance
